# Materia Medica and Therapeutics

**Published:** 1896-09

**Authors:** J. S. Cassidy

**Affiliations:** Covington, Ky.


### Materia Medica and Therapeutics.

BY J. S. CASSIDY, D.D.S., M.D., COVINGTON, KY.

Reported to the American Dental Association, Saratoga, N. Y., August, 1896.

The process of cataphoresis has probably been the most interesting new feature of dental therapeutics introduced during the past year. Cataphoretic medication, however, has been unconsciously practiced, to a limited extent, for several years, by what was supposed to be merely the effects of the electrolysis of certain drugs in the local treatment of alveolar pyorrhœa, and other septic conditions pertaining to the teeth.

At the clinics of the dental section of the International Medical Congress, at Washington, in 1887, Dr. W. V. B. Ames exhibited a simple electric appliance for developing active iodin at the desired point by the electrolysis of KI. At the same clinic, another gentleman, whose name the writer has forgotten, adopted similar means with a solution of NaCl to bleach darkened teeth. This statement is made merely with a view to the recognition of evolution in this phase of electro-therapeutics, and not for the purpose of diminishing the credit due to Prof. Morton, and others, for introducing this new osmotic treatment.

The physiological benefits obtained in the majority of cases, through cataphoresis, are accepted facts, but the phenomena involved in their production, as yet, remain unexplained.

Heretofore the effects of electric currents on compound liquids have been studied only in connection with electrolysis, and the questions might be asked,

Does cataphoresis ever occur free from electrolysis? Yes.

Does electrolysis, of an electrolyte in porous solid or semi-solid matter, always accompany cataphoresis?

To this I would answer; No, as witness the passage of water in moistened clay toward the cathode without suffering decomposition, and, conversely, the decomposition of KI in contact with gum-tissue, where no intromission of the freed iodin is apparent. This latter example seems to prove that at least one of the laws of ordinary electrolysis might be applied with advantage, in many cases, to our choice of drugs. For instance, those whose decomposition will supply its electro-positive radical as the active agent, will penetrate more deeply; otherwise, as in the case of KI the electro-negative radical I, will not be drawn or pushed cataphorically toward the negative pole; but when free iodin in aqueous solution is used, it acts most charmingly, owing to the carrying power of the water, absence of chemical action, and consequent non-interference with its passage by a more highly electro-positive radical. The extra energy noticed in bleaching, of electro-negative O, developed by this process from aqueous pyrozone, may be accounted for by its greatly increased volume and pressure, due to the electrolysis.

Electro-negative radicals are attracted toward the anode and the positive radicals to the cathode, probably somewhat according to the following arrangement of KI:

K K K K K K—

+I I I I I I

Dr. L. P. Bethel has reported, recently, successful applications of this method in introducing solution of $AgNo_3$ into root-canals; the canal, in each instance, although too small for any broach to enter, was completely permeated. This statement is not surprising, however, if we consider the nature of the substance. It decomposes into basic and acid radicals by contact with organic matter, even when applied topically; it is a good electrolyte, and, therefore, the influence of the electric current causes further progression of the basic radical $Ag_2O$ than would occur did separation not take place. The silver oxid deposits as an insoluble, black, inert residue, and the acid radical of the salt escapes with water as nitric acid:

$$2AgNo_3 = Ag_2O + N_2O_5.$$

$$N_2O_5 + H_2O = 2HNO_3.$$

One other rule of electrolysis may be alluded to in this connection, namely: "decomposition of an electrolyte is in the ratio of its chemical equivalent." Accordingly 9 parts by weight $H_2O$, 98 of $H_2SO_2$, and 166 of KI, will react simultaneously by the influence of the same current.

This rule may enable some one to construct a list of—may I venture to name them? "*Cataphoretics*," by combining in certain proportions a too active conductor with a less active one, as in the case of cocain hydrochlorid and guaiacol.

Objections have been made to the use of sulfuric acid by this method, on account of its loss of power by electrolysis. This, however, is not practically true, for although it decomposes into free H, and sulfione, it is again reconstructed at the expense of the water present, enabling the nascent molecule to attack the dentine with more then usual energy, at the same time setting O free.

$$H_2SO_4 = H_2 + SO_4$$

$$SO_4 + OH_2 = H_2SO_4 + O.$$

A liquid conductor, more or less above zero, seems to be essential to this process, and different preparations of the same active principle are indicated for the variations in treatment. As is already well known, the action of cocain hydrochlorid is more effective in reducing the sensibility of dentine, if it be retarded by a non-conductor, such as some of the phenols, of which guaiacol, thus far, is preferred. But when using this salt as a local anæsthetic for soft tissues, a strong aqueous solution would be my own preference.

Dr. Custer uses blotting-paper, saturated with the solution, and of a size corresponding to the area to be anæsthetized; he places the pad in position, a silver coin is then interposed between it and the anode, and the current turned on. For soft tissues both voltage and amperage may be increased above that indicated for sensitive dentine, without producing any unpleasant effects.

In preparing for extracting teeth, a forked anode, adjusted by a ball and socket-joint, should be used, one prong placed in the cavity of decay, if there be such, with cocain held by cotton wool, and the other point placed well up on the gum in the manner described by Dr. Custer. Dr. Custer further writes: "I am satisfied on the following: Cocain, cataphorically applied on the mucous membrane—and upon the cuticle as well—is almost, if not quite, as effective as a hypodermatic injection. It also possesses these advantages:

"First: The application itself, if properly conducted, is painless.

"Second: There is less danger, because the drug is diffused, and it is not possible for the full dose to enter into the venous circulation, as may happen when administered by the syringe. (I believe all the cases of sudden collapse by injection are due to the dose being injected into a vein and thereby carried, undiluted, to the heart and medulla reflexly.) It is when the cocain is mostly entered into the arteries that the anæsthesia is best and the danger least.

"Third: The danger of septic inoculation is overcome."

A few weeks ago the attention of the writer was called to the decided acid reaction produced by cataphoresis of cocain hydrochlorid on sensitive dentine. This reaction is due to electrolysis of the salt, the alkaloid proceeding in search of its beloved cathode, while the acid H Cl remains at the surface near its own favorite anode. At first this condition suggested the presence of an enemy, but later experiments proved that the relatively small quantity of acid developed can be of no serious consequence to the dentine.

A new remedy has made its appearance recently, bearing the name of eucain, which will probably soon claim a large share of the honors hitherto belonging to cocain as a local anæsthetic. It is an artificial alkaloid, said to be produced by the reaction between, principally, acetone (dimethyl ketone, $CH_3COCH_3$) and ammonia. The favorite salt is, like that of cocain, the hydrochlorid, which, evaporated from aqueous solution, retains one molecule of water of crystallization, $C_{19}H_{27}NO_4HClH_2O$. Eucain hydrochlorid presents the appearance of a white, odorless, crystalline powder, of a bitter, quinin-like taste, soluble in water, alcohol and chloroform. It induces some hyperæmia of the mucosæ, rather than anæmia, when applied locally, but nevertheless, it causes well-marked loss of sensibility. As a "cataphoretic" obtundent of dentine it seems to act favorably, although more experiments will have to be made with it before its virtues in this direction are accepted as satisfactory. From the literature accompanying the sample sent me I learn that eucain is less poisonous than cocain, although their dosages are approximately the same; that lethal doses cause excitation of the central nervous system, convulsions affecting all the muscles, general paralysis, and, finally, death of the animal by respiratory failure. For producing local anæsthesia by hypodermatic injection it is advised to make a solution of one (1) part of eucain hydrochlorid to ten (10) parts of sterilized water. Such a solution, it is claimed, remains permanently unchanged, and can be boiled without causing decomposition. In this particular, at least, it has a decided advantage over cocain; it is probably uniform in its action, other conditions being equal, on account of its immediate derivation from pure chemicals, instead of indirectly from vegetable sources. The claim is made, further, that no evil effects have thus far been reported as sequelæ of hypodermatic injection for the removal of teeth, although the anæsthesia, in point of duration and intensity, was fully as satisfactory as that produced by cocain.

While acknowledging that there must be differences in degrees of danger by the use, and sometimes abuse, of different drugs introduced by the needle, the evidence of experience proves that unpleasant consequences may follow such an operation, even when the most innocent of all substances be the only one employed.

